# Ethnicity and Smoking-Associated DNA Methylation Changes at HIV Co-Receptor GPR15

**DOI:** 10.3389/fpsyt.2015.00132

**Published:** 2015-09-22

**Authors:** Meeshanthini V. Dogan, Jinhua Xiang, Steven R. H. Beach, Carolyn Cutrona, Frederick X. Gibbons, Ronald L. Simons, Gene H. Brody, Jack T. Stapleton, Robert A. Philibert

**Affiliations:** ^1^Department of Biomedical Engineering, University of Iowa, Iowa City, IA, USA; ^2^Department of Psychiatry, University of Iowa, Iowa City, IA, USA; ^3^Department of Internal Medicine, University of Iowa, Iowa City, IA, USA; ^4^Iowa City Veterans Affairs, Iowa City, IA, USA; ^5^Center for Family Research, University of Georgia, Athens, GA, USA; ^6^Department of Psychology, Iowa State University, Ames, IA, USA; ^7^Department of Psychology, University of Connecticut, Storrs, CT, USA

**Keywords:** epigenetics, smoking, GPR15, AHRR, African Americans, European Americans, HIV, ethnicity

## Abstract

Smoking is associated with poorer health outcomes for both African and European Americans. In order to better understand whether ethnic-specific genetic variation may underlie some of these differences, we compared the smoking-associated genome-wide methylation signatures of African Americans with those of European Americans, and followed up this analysis with a focused examination of the most ethnically divergent locus, cg19859270, at the *GPR15* gene. We examined the association of methylation at this locus to the rs2230344 SNP and *GPR15* gene and protein expression. Consistent with prior analyses, AHRR residue cg05575921 was the most differentially methylated residue in both African Americans and European Americans. However, the second most differentially methylated locus in African Americans, cg19859270, was only modestly differentially methylated in European Americans. Interrogation of the methylation status of this CpG residue found in GPR15, a chemokine receptor involved in HIV pathogenesis, showed a significant interaction of ethnicity with smoking as well as a marginal effect of genotype at rs2230344, a neighboring non-synonymous SNP, but only among African Americans. Gene and protein expression analyses showed that demethylation at cg19859270 was associated with an increase in both mRNA and protein levels. Since GPR15 is involved in the early stages of viral replication for some HIV-1 and HIV-2 isolates, and the prevalence of HIV is increased in African Americans and smokers, these data support a possible role for GPR15 in the ethnically dependent differential prevalence of HIV.

## Introduction

Smoking is the largest preventable cause of morbidity and mortality in the United States (1). Each year, nearly half a million Americans die from the complications of smoking. Smoking exerts its effects by increasing mortality through more “traditional” disorders such as chronic obstructive pulmonary disease (COPD), heart disease, and stroke.

In efforts to understand sources of increased risk for mortality through these diseases and other smoking-associated disorders, a large number of genome-wide association studies (GWAS) of genetic variation have been conducted (2–5). Despite the strong heritable contribution to many of these complex disorders, GWAS have identified only a small fraction of the total genetic component. This lack of success in identifying the “hidden genetic variation” underlying these illnesses limits our ability to translate sequence-based findings into tools with significant impact on treatment or diagnosis. Although there may be many reasons for these failures, one commonly cited problem is the confounding of potential genetic signals by gene–environment interactions (GxE) (6, 7). One of the important confounders may be smoking (8).

The failure to understand the genetic architecture of these smoking-related illnesses is a substantial problem. Unfortunately, like many illnesses, the burden of smoking-related illness is not shared equitably. For example, with respect to ethnicity, even after adjustment for the usual covariates such as access to health care, African Americans are more likely than European Americans to suffer adverse outcomes for many smoking-related illnesses such as lung cancer and heart disease (9, 10). Subtle or non-additive genetic differences may underlie these differential vulnerabilities. The challenge lies in establishing a research paradigm through which sources of hidden genetic variance can be more readily identified and the search narrowed.

Ironically, an improved understanding of genetic contributions to smoking-related illnesses may come about through a better understanding of the epigenetic influences of smoking on the genome. In early 2012, we demonstrated that smoking is associated with genome-wide changes in DNA methylation, with the most sensitive changes being noted at the aryl hydrocarbon receptor repressor (*AHRR*) (11). These findings have been extensively replicated with demethylation at *AHRR* CpG residue cg05575921 being consistently the top or second most prominent change in eight consecutive independent genome-wide analyses of European and African Americans of all ages (12–19). However, not all residues show such consistent change in response to smoking. In particular, cg19859270, a residue in G-protein-coupled receptor 15 (*GPR15*), is not significantly differentially methylated early in new-onset smokers, and in our cursory review of genome-wide methylation studies of adult smokers, seemed to be more differentially methylated in studies of African Americans compared with those of European Americans.

*GPR15* is a particularly intriguing candidate for contributing to “hidden” variance for smoking-associated illness. This small chromosome 3 gene (1254 bp mRNA) codes for a chemokine receptor (a.k.a. Brother of Bonzo or BOB) that acts as a co-receptor for human immunodeficiency virus (HIV) type 2 and is capable of serving as a co-receptor for some HIV-1 isolates (20–22). These observations are relevant to the identification of hidden variance for complex illness for two reasons. First, even after controlling for exposure and health care accessibility variables, African Americans are more likely to be infected with HIV and die from smoking-related cancer and coronary artery disease (CAD) than European Americans (23). Second, smoking is a major risk factor for these illnesses. When taken together, these observations suggest that *GPR15* may be an ideal locus through which to develop an understanding of how hidden genetic variance may interact with environmental factors, such as tobacco smoke, in fulminating illness.

Therefore, in this study, we extend our prior study of adult female African Americans by conducting additional genome-wide methylation analyses of male European and African Americans. We then describe an integrated set of follow-up experiments at the locus with the most discrepant methylation changes to investigate whether ethnic contextual effect of smoking on cg19859270 (*GPR15*) methylation is driven by local or non-local SNP variation.

## Materials and Methods

### Informed consent

Participants recruited for this study provided written consent to participate in this study. All protocols and procedures pertaining to Family and Community Health Study (FACHS) were approved by the Institutional Review Board at the University of Iowa, the University of Georgia, and Iowa State University. All protocols and procedures pertaining to the Behavioral Diagnostics (BD) study were approved by the Institutional Review Board at the University of Iowa.

### Genome-wide DNA methylation

The genome-wide analysis included 100 adult males from the FACHS and a study of substance use performed in collaboration with a commercial company, BD (24). Clinical data for the analysis were obtained from structured interviews with an adapted form of the Semi-Structured Assessment for the Genetics of Alcoholism-II (SSAGA-II) (25). Active smokers were classified as smokers while individuals denying use of any tobacco products were classified as non-smokers. Subjects were also phlebotomized to obtain biomaterial. Characteristics of these subjects are presented in Table 1. Briefly, of these 100 middle-aged individuals, 48 were European Americans and 52 were African Americans. Nineteen European Americans and 24 African Americans were smokers.

**Table 1:** **Clinical and demographic characteristics of male subjects participating in the genome-wide methylation studies by ethnicity**.

	European Americans		African Americans	
	Smoker	Control	Smoker	Control
Age	45 ± 8	47.1 ± 8	49.6 ± 6	49.5 ± 7
Smoking status	19	29	24	28
Current average cigarettes/day	21 ± 11		11 ± 6	
Smoker pack-years^a^				
≥1/2 and <10	1		12	
≥10 and <20	3		6	
≥20	12		6	

*^a^Total number of smoking individuals does not match the number of individuals accounted for under pack-years due to missing data points*.

Peripheral mononuclear cell pellets were obtained through standard Ficoll purification, and DNA was subsequently extracted using standard manufacturer protocol provided in the Qiagen (Valencia, CA, USA) DNA Mini Kit. The Illumina (San Diego, CA, USA) HumanMethylation450 Beadchip was used to profile the genome-wide DNA methylation status across 485,577 CpG loci of subjects in this study. This was conducted by the University of Minnesota Genome Center (Minneapolis, MN, USA). Details of the procedure have been described previously (18).

The beta value at a CpG locus is defined as the ratio between the methylated probe intensity and the total intensities of methylated and unmethylated probes. The average beta value at each locus was determined using the Illumina Genome Studio Methylation Module Version 3.2, and values with a detection *p*-value greater than 0.05 were removed using a PERL script.

The genome-wide data were analyzed using the MethLAB Version 1.5 (26), an R package, as previously described (13). Smoking-related differential methylation between smokers and non-smokers was assessed while controlling for ethnicity, slide, and plate effects. Correction for multiple comparisons was conducted using the method of Benjamini–Hochberg in MethLAB at the 0.05 significance level (27). Likewise, a similar genome-wide analysis was performed separately to determine the differential methylation of each ethnicity. For this analysis, the genome-wide DNA methylation was analyzed with respect to smoking status while controlling for slide and plate effects.

### Genotyping rs2230344

In order to test if the effect of smoking on cg19859270 (*GPR15*) methylation is ethnically or genetically contextual, we genotyped the only common non-synonymous sequence polymorphism (rs2230344) in the immediate proximity of cg19859270. Since there is no effect of gender on methylation at the cg19859270 locus (data not shown), in order to increase the power of our analyses, we also profiled this genotype in the female subjects from the FACHS (*n* = 111) and BD (*n* = 14) cohorts. This increased our sample size from 100 to 225. Whole blood DNA from these individuals was genotyped for the rs2230344 SNP using the SNP Genotyping Assay (Life Technologies, Carlsbad, CA, USA) and standard manufacturer protocol. Samples alongside the genotyping solution were plated in a 384-well plate. Genotyping at rs2230344 was conducted as previously described using reagents from Applied Biosystems (Foster City, USA) (24). Of the 225 individuals, 219 were successfully genotyped. The genotype was coded as either 0 for the absence of the minor *T* allele (i.e., *C,C* genotype) or 1 for the presence of at least one minor *T* allele (i.e., *C,T* and *T,T* genotypes).

### Real-time PCR for gene expression quantification

Of the 219 successfully genotyped individuals, 121 African American subjects had lymphoblast cell lines that were available for gene expression profiling. RNA from these subjects extracted from lymphoblast cell lines using the standard manufacturer protocol of PureLink RNA Mini Kit (Life Technologies) was used for *GPR15* gene expression profiling. RNA from these samples was reverse transcribed to cDNA using standard manufacturer protocol of the High-Capacity cDNA Reverse Transcription Kit (Life Technologies). After conversion, samples were loaded in duplicates in a 384-well plate alongside two housekeeping genes, *LDHA* and *RPL7A*. The TaqMan gene expression assays were used (Life Technologies). Gene expression profiling was conducted in an ABI 7900HT Fast Real-Time PCR system (Life Technologies), and the resulting Ct values were exported. *GPR15* Ct values were normalized using the Ct geometric mean values of *LDHA* and *RPL7A* housekeeping genes.

### Western blot for protein quantification

Peripheral blood mononuclear cell pellets from 10 individuals were used: five with highest and five with lowest methylation at cg19859270. Cells were treated in lysis buffer (Cell Signaling Technology, Beverly, MA, USA) for 15 min and sonicated briefly. Lysates were separated by polyacrylamide gel electrophoresis and transferred to a nitrocellulose membrane. Membranes were incubated in protein-free blocking buffer (Thermo Fisher Scientific, Waltham, MA, USA) for 1 h at room temperature, followed by incubation with primary antibody (1:1000 dilution). Immunoreactive proteins were detected with Amersham ECL (GE Healthcare, Pittsburgh, PA, USA) using a Kodak Imager. Images were analyzed by ImageJ software (NIH).

### Regression analyses

All regression analyses of methylation at cg19859270, rs2230344 genotype, and *GPR15* gene expression were conducted in JMP Version 9 (SAS Institute, Cary, NC, USA).

## Results

### Genome-wide DNA methylation analyses

Table 1 describes the key demographic characteristics of the 100 male subjects from the FACHS and BD cohorts whose data are included in the genome-wide contrasts of smoking status. In total, peripheral mononuclear cell (a.k.a. PBMC or lymphocyte pellet) DNA from 52 African Americans (24 smokers, 28 non-smokers) and 48 European Americans (19 smokers and 29 non-smokers) were included in the genome-wide analysis. The European American subjects were slightly younger and smoked more cigarettes on average in a day.

As a first step, the genome-wide methylation data from all male subjects (*n* = 100) were analyzed together with respect to smoking status, controlling for ethnicity, slide, and plate effects. In this initial analysis, a total of 404 CpG loci were significantly differentially methylated at the 0.05 level after Benjamini–Hochberg correction for multiple comparisons. The 30 most significant loci are shown in Table 2.

**Table 2:** **The 30 most significantly associated probes with respect to smoking status after genome-wide correction**.

Probe ID	Gene	Placement	Island status	Average beta values		*T*-test	Corrected *p*-value
				Smokers	Non-smokers		
cg05575921	AHRR	Body	N_Shore	0.67	0.85	5.31E−18	2.58E−12
cg01940273			Island	0.49	0.59	7.85E−14	1.91E−08
cg05951221			Island	0.29	0.38	4.69E−13	7.59E−08
cg21566642			Island	0.36	0.47	8.77E−13	1.07E−07
cg03636183	F2RL3	Body	N_Shore	0.54	0.66	1.70E−11	1.65E−06
cg19859270	GPR15	1st Exon		0.79	0.85	7.61E−10	6.16E−05
cg23576855	AHRR	Body	N_Shore	0.50	0.67	1.71E−09	1.13E−04
cg26703534	AHRR	Body	S_Shelf	0.62	0.68	1.86E−09	1.13E−04
cg03329539			N_Shore	0.32	0.37	3.11E−09	1.68E−04
cg06126421				0.64	0.74	4.01E−09	1.95E−04
cg09741592	HNRNPA1	Body	S_Shore	0.21	0.24	2.43E−08	1.07E−03
cg10190813	HUS1	Body	N_Shore	0.13	0.16	3.90E−08	1.58E−03
cg21161138	AHRR	Body		0.62	0.68	9.90E−08	3.70E−03
cg02657160	CPOX	Body	N_Shore	0.77	0.81	2.17E−07	7.53E−03
cg08709672	AVPR1B	5′UTR	S_Shore	0.63	0.66	2.40E−07	7.78E−03
cg25114611	FKBP5	TSS1500	S_Shore	0.28	0.32	2.74E−07	8.31E−03
cg11554391	AHRR	Body	Island	0.16	0.20	3.15E−07	8.68E−03
cg14656043	CREM	5′UTR	S_Shelf	0.18	0.22	3.22E−07	8.68E−03
cg02767841	ZMYND8	TSS1500		0.09	0.11	4.06E−07	1.01E−02
cg15794034	AMICA1	1st Exon		0.07	0.10	4.14E−07	1.01E−02
cg04267000	EHMT2	Body		0.98	0.97	4.97E−07	1.15E−02
cg16451872	UBE3A	TSS1500	S_Shore	0.18	0.22	5.50E−07	1.17E−02
cg03450842	ZMIZ1	5′UTR		0.55	0.60	5.54E−07	1.17E−02
cg17580614	ADORA2B	Body	Island	0.69	0.74	7.61E−07	1.53E−02
cg05649724			Island	0.22	0.26	7.87E−07	1.53E−02
cg09336260	GPR34	1st Exon		0.19	0.24	9.35E−07	1.70E−02
cg08170227	ACTN1	Body		0.48	0.52	9.67E−07	1.70E−02
cg05003417	NARF	5′UTR	Island	0.05	0.06	9.84E−07	1.70E−02
cg01692968			N_Shore	0.34	0.40	1.02E−06	1.70E−02
cg13245152	PAX6	Body	S_Shore	0.25	0.28	1.10E−06	1.78E−02

*All average methylation values are non-log-transformed beta values. Island status refers to the position of the probe relative to the island. Classes include (1) Island, (2) N (north) shore, (3) S (south) shore, (4) N (north) shelf, (5) S (south) shelf, and (6) blank denoting that the probe does not map to an island*.

Consistent with our prior genome-wide analysis conducted on FACHS females (18), probes cg05575921 (ranked first in males and second in females) and cg23576855 (ranked seventh in males and fifth in females) from the *AHRR* gene and cg19859270 (ranked sixth in males and first in females) from the *GPR15* gene were highly ranked. However, the cg03636183 probe (ranked fifth in males) from the coagulation factor II (thrombin) receptor-like 3 (*F2RL3*) gene is apparent in male but not female smokers (18). On average, at these four highly ranked loci in the males, smokers tended to be consistently hypomethylated which conventionally is attributed to increased gene expression.

Because of our informal observations and the work of Elliott and colleagues who have also noted discrepancies between ethnic groups (19), we next analyzed genome-wide DNA methylation with respect to smoking status for each ethnic group. The results for the smaller European American cohort were less robust. After correction for genome-wide contrasts, only one locus, *AHRR* residue cg05575921, was significantly differentially methylated. A list of the 30 most significant loci and the average methylation for the cases and controls are shown in Table 3.

**Table 3:** **The 30 most significantly differentially methylated probes with respect to smoking status after genome-wide correction in European Americans (EA) and the corresponding probe ranking in African Americans (AA)**.

Probe ID	Gene	Placement	Island status	Rank in AA	AA		EA	
					Average beta values		Average beta values	
					Smokers	Non-smokers	Smokers	Non-smokers
cg05575921	AHRR	Body	N_Shore	1	0.65	0.80	0.70	0.90
cg01940273			Island	8	0.48	0.58	0.50	0.60
cg04359418			Island	382,980	0.82	0.82	0.81	0.87
cg21475150	RPL31	TSS1500	Island	388,413	0.84	0.85	0.78	0.84
cg15633035	IL6R	3′UTR		117,278	0.33	0.37	0.34	0.42
cg21566642			Island	9	0.37	0.48	0.35	0.46
cg05951221			Island	7	0.28	0.37	0.31	0.40
cg17179314	CHRNB4	TSS1500	Island	351,840	0.60	0.61	0.55	0.60
cg03827423	ISCU	TSS1500	N_Shore	441,480	0.03	0.03	0.03	0.05
cg17581065	CCDC42	Body		426,434	0.94	0.94	0.91	0.92
cg22888484	SNHG11	TSS200	N_Shore	271,892	0.07	0.06	0.04	0.06
cg19731612	NSD1	TSS1500	Island	206,301	0.71	0.73	0.63	0.76
cg17367884	TBC1D16	Body	Island	307,634	0.80	0.79	0.85	0.87
cg03114244	FEN1	5′UTR	Island	53,788	0.02	0.03	0.01	0.02
cg23193759	C10orf35	TSS200	Island	86,696	0.16	0.18	0.13	0.16
cg16568472	ITPA	Body	S_Shore	231,862	0.15	0.05	0.05	0.05
cg26703534	AHRR	Body	S_Shelf	333	0.62	0.67	0.63	0.69
cg04075184	CLASP1	Body	S_Shelf	58,660	0.69	0.72	0.70	0.75
cg02004401	GNA12	3′UTR		249,450	0.65	0.70	0.71	0.78
cg00476430	PAG1	5′UTR		75,788	0.77	0.80	0.82	0.79
cg01760846	PSMC1	TSS200	N_Shore	342,153	0.17	0.17	0.17	0.21
cg10068330				244,013	0.60	0.61	0.69	0.64
cg21053365	ZNF425	Body	N_Shore	363,555	0.05	0.05	0.05	0.06
cg16168311	APOA1BP	Body	S_Shore	36,107	0.21	0.23	0.12	0.14
cg14519950	C9orf68	Body	Island	29,587	0.05	0.06	0.05	0.06
cg10200202	RGS10	Body		44,756	0.08	0.09	0.07	0.09
cg10755899			Island	70,761	0.81	0.82	0.81	0.87
cg12864389	PLEC1	Body	Island	41,579	0.20	0.22	0.20	0.26
cg13910681	FAM102A	Body	N_Shore	17,620	0.15	0.17	0.14	0.18
cg04799493				5,613	0.92	0.90	0.91	0.90

In contrast, for the larger African American cohort, after genome-wide correction, 84 CpG loci remained significantly differentially methylated at the 0.05 level. A list of the 30 most significant loci and the average methylation in the case and control samples are shown in Table 4.

**Table 4:** **The 30 most significantly differentially methylated probes with respect to smoking status after genome-wide correction in African Americans (AA) and the corresponding probe ranking in European Americans (EA)**.

Probe ID	Gene	Placement	Island status	Rank in EA	AA		EA	
					Average beta values		Average beta values	
					Smokers	Non-smokers	Smokers	Non-smokers
cg05575921	AHRR	Body	N_Shore	1	0.65	0.80	0.70	0.90
cg19859270	GPR15	1st Exon		26,702	0.78	0.86	0.80	0.84
cg23576855	AHRR	Body	N_Shore	4,194	0.50	0.68	0.52	0.65
cg09741592	HNRNPA1	Body	S_Shore	75,481	0.19	0.23	0.25	0.26
cg02657160	CPOX	Body	N_Shore	57,537	0.77	0.81	0.77	0.79
cg03636183	F2RL3	Body	N_Shore	154	0.52	0.65	0.58	0.68
cg05951221			Island	7	0.28	0.37	0.31	0.40
cg01940273			Island	2	0.48	0.58	0.50	0.60
cg21566642			Island	6	0.37	0.48	0.35	0.46
cg23254918	CD59	TSS200	Island	113,560	0.05	0.06	0.05	0.05
cg06620353	SEC63	Body		284,374	0.93	0.90	0.78	0.77
cg01866630	ITPR3	Body	Island	328,999	0.09	0.10	0.08	0.07
cg04120842	PARP4	Body		295,071	0.96	0.95	0.89	0.89
cg25937832	NKIRAS1	5′UTR	Island	165,126	0.05	0.06	0.06	0.06
cg10486879	NCRNA00167	Body	Island	381,424	0.03	0.03	0.03	0.03
cg26040729	RSF1	Body	S_Shore	205,668	0.91	0.89	0.74	0.75
cg26701198	COG6	TSS200	N_Shore	75,566	0.07	0.08	0.07	0.08
cg23028848	TNKS1BP1	TSS200	Island	107,426	0.03	0.04	0.04	0.04
cg01345087	SYN1	5′UTR	Island	153,964	0.04	0.05	0.04	0.08
cg07904448	ZNF529	5′UTR	Island	201,311	0.06	0.07	0.06	0.06
cg11835582	FBXO8	1st Exon	Island	364,823	0.04	0.05	0.04	0.04
cg03904534	WWOX	Body		306,400	0.98	0.97	0.94	0.94
cg04267000	EHMT2	Body		55,247	0.98	0.97	0.98	0.98
cg02632643	CD320	Body	N_Shelf	228,457	0.96	0.95	0.96	0.95
cg14405773	CSH2	Body		272,998	0.98	0.98	0.97	0.97
cg26858409	ZFP64	Body	S_Shore	90,596	0.89	0.90	0.88	0.89
cg19111030	ANKRD53	TSS1500	N_Shore	350,384	0.18	0.20	0.18	0.19
cg23093692	SLC35F3	Body	Island	94,323	0.79	0.73	0.74	0.70
cg11556164	LRRN3	5′UTR		231,425	0.70	0.77	0.70	0.72
cg22949575	ASPSCR1	Body	N_Shelf	286,780	0.97	0.96	0.94	0.94

Comparison and contrast of the relative ranking of the findings of the two groups were illuminating. Consistent with our observations formed by the review of the previous literature, *GPR15* residue cg19859270 (ranked second in African American males) was the 26,702nd ranked probe in the European American cohort (see Table 4). Also, the cg03636183 locus from the *F2RL3* gene was highly ranked in African Americans (ranked sixth) and not in the European Americans (ranked 154th). In fact, outside the *AHRR* (cg05575921) and the three intergenic loci (cg05951221, cg01940273, and cg21566642), which map to a CpG island rich in transcription factor motifs approximately 9 kb upstream of the *ALPPL2* locus, there seems to be little correspondence in the relative rankings of the probes between the two cohorts, suggesting the possibility that ethnic-specific genetic variation may be influencing the degree of smoking-associated DNA methylation at a majority of loci.

### Focus on GPR15

**Figure 1:**
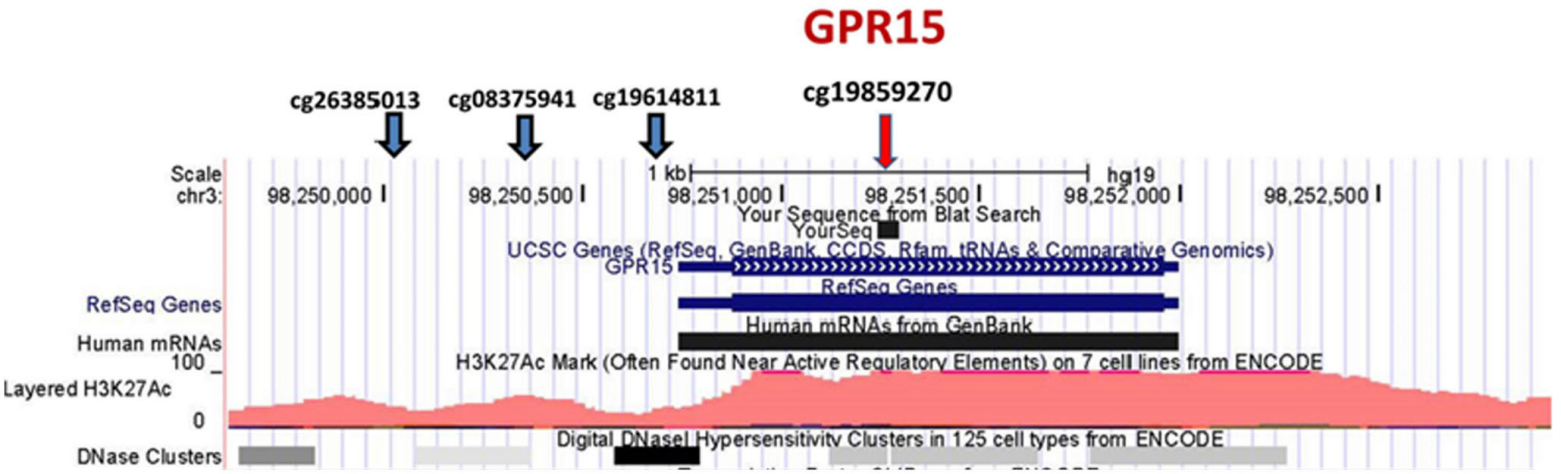
**Position of Illumina GPR15 methylation probes**. The position of Illumina *GPR15* methylation probes per the UCSC Genome Browser (http://genome.ucsc.edu). The genome-wide corrected *p*-values for the CpG residue probes are *p*-value <0.90, *p*-value <0.39, *p*-value <0.10, and *p*-value <6.2E−05, respectively (left to right).

Since our initial analyses seemed to confirm our prior impressions that *GPR15* was more prominently differentially methylated in African American smokers, we focused our inquiry on *GPR15*. Figure 1 illustrates key features of this chromosome 3 locus. *GPR15* is a single exon gene coding for 1254-bp mRNA and a 360 amino acid protein. Overall, the gene region is sparsely populated with CpG residues (19 CpG residues in the 2300-bp region covering the gene region). There are four probes in the Illumina 450K array covering *GPR15*, cg26385013 (TSS1500), cg08375941 (TSS1500), cg19859270 (first Exon), and cg19614811 (TSS200). In contrast to the strong signal at cg19859270, methylation at the other three *GPR15* CpG residues is not significantly associated with smoking status [cg26385013 (*p*-value <0.90), cg08375941 (*p*-value <0.39), and cg19614811 (*p*-value <0.10)].

The rs2230344 polymorphism was chosen because it is the only common sequence polymorphism in the immediate proximity of cg19859270 (310 bp upstream). Review of the dbSNP database shows this to be a *C* to *T* polymorphism resulting in a Proline or Serine, respectively, at amino acid 37 of *GPR15*. According to the dbSNP database, the minor allele frequency (MAF) is approximately 0.16 in European Americans and 0.05 in Yorubans (28). Using standard methods, we then determined genotype at this locus for all subjects. In our European American male subjects, the MAF was 0.23 while in our African American male subjects the MAF was 0.06.

### Effect of age, gender, and smoking consumption history on cg19859270 methylation

As part of the initial inquiry into other variables that may be influencing the degree of methylation at *GPR15* residue cg19859270, we first examined whether the age/total smoking consumption and gender of the smoker might significantly influence methylation at cg19859720. Fortunately, in prior work, our group has conducted genome-wide analyses of smoking in 19-year-old African Americans (both genders, *n* = 399), 22-year-old African Americans (males only *n* = 107), and adult female African Americans (*n* = 111) using the Illumina 450 K platform (13, 16, 18). We used these data in combination with the current data set of 100 males to understand the effects of age, gender, and smoking exposure. In our contrast of 19- and 22-year-old smokers who averaged 1/2 and 1 pack year of consumption, respectively, the difference in methylation between the cases and controls at cg19859270 is 0.01 (nominal *p*-value <0.002, rank 863) and 0.02 (nominal *p*-value <0.0006, rank 164), respectively. Finally, using the combined data from the current male and previously examined female African Americans, we examined the effect of gender on methylation and found no significant effect (*p* < 0.9). Taken as a whole, the data indicate that the changes in cg19859270 methylation are incremental and dose dependent, but not grossly influenced by gender.

### Effect of smoking, ethnicity, and genotype on cg19859270 methylation

As the next step, we tested whether the effect of smoking on cg19859270 methylation is ethnically or genetically contextual. To do this, we constructed a model stipulating methylation at cg19859270 as the dependent variable, with ethnicity, rs2230344 genotype, smoking status, and their interactive terms (e.g., ethnicity × smoking status) as independent variables.

Overall, the model was highly significant with an adjusted *R*^2^ of 0.54 (*p*-value <0.0001). Smoking status had a highly significant main effect [sum of squares (SS) = 0.16, *p*-value <0.0001], a trend for a main effect of genotype (SS = 0.005, *p*-value <0.06) with no significant main effects of ethnicity (SS = 0.0004, *p*-value <0.59). The interaction term between ethnicity and smoking status was significant (SS = 0.013, *p*-value <0.002), but there were no effects for the other three interaction terms. In short, the model supports the hypothesis that factors segregating with ethnicity moderate the effects of smoking on DNA methylation at cg19859270.

Because of the interaction effect of ethnicity with smoking status, we then tested the relationship of rs2230344 genotype to cg19859270 methylation for each ethnicity using both smoking status and genotype as independent variables and incorporating an interaction term of genotype with smoking status. The overall models were highly significant for both groups with the proportion of the variance accounted for by the model for African Americans (adjusted *R*^2^ = 0.61, *p*-value <0.0001) being considerably larger than that for European Americans (adjusted *R*^2^ = 0.20, *p*-value <0.002). In both models, smoking was the largest predictor of cg19859270 methylation (*p*-value <0.0001). However, in contrast to the European Americans, there was a significant main effect of rs2230344 genotype in the African American subjects (*p* < 0.05), suggesting that methylation level in African Americans is affected not only by smoking status but also by the SNP. On the contrary, methylation in European Americans is only affected by smoking status.

### Effect of smoking status and rs2230344 SNP genotype on GPR15 RNA and protein levels

In previous work, we have shown that smoking-associated changes in peripheral blood DNA are reflected, but attenuated in lymphoblast cell lines prepared from those blood cells (11). Because alterations in DNA methylation are associated with changes in gene expression and there is evidence for the effect of rs2230344 genotype on methylation only in African Americans, we next examined whether rs2230344 genotype was associated with changes in *GPR15* gene expression using RNA from lymphoblast cell lines prepared from 121 African American participants characterized for smoking status (81 non-smokers and 40 smokers) and enriched for the rs2230344 *T* allele (95 *CC*, 23 *CT*, and 3 *TT*) using a general linear modeling approach. Genotypes were grouped into two groups: absence of minor *T* allele and presence of at least one minor *T* allele. Overall, the model that included genotype, smoking status, and their interaction term accounted for 9% of the variance (adjusted *R*^2^ = 0.086, *p*-value <0.004) with significant main effects of smoking status (SS = 2.98, *p*-value <0.03) and a trend toward significance for both genotype (SS = 1.72, *p*-value <0.10) and the interaction term (SS = 2.15, *p*-value <0.07).

**Figure 2:**
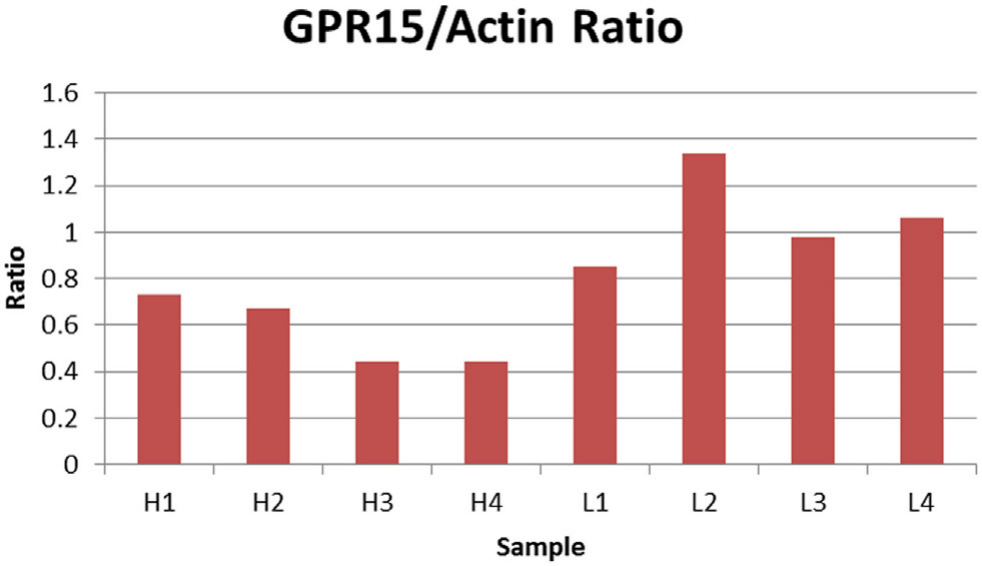
**Relative ratio of GPR15/Actin**. The figure depicts the relative ratio of GPR15/Actin for each PBMC cell pellet. H1 through H4 represent the four samples with the highest cg19859270 methylation, whereas L1 through L4 represent four samples with the lowest cg19859270 methylation.

Finally, we examined whether cg19859270 methylation was associated with GPR15 protein expression. We utilized lymphocyte pellets from the BD cohort to determine the extent to which protein expression was influenced by methylation at cg19859270. The relative level of GPR15 protein was measured in 10 individuals, five with the highest and five with the lowest methylation levels at cg19859270. Results from two failed samples were excluded from the analysis (one high and one low methylation). Protein levels normalized to actin housekeeping protein was inversely correlated with methylation (*p*-value <0.01). Figure 2 illustrates the results. As can be seen, all four of the less methylated cells show greater relative production of GPR15 protein than any of the cells with a high level of methylation, indicating a substantial association between protein production and gene methylation at cg19859270. Figure S1 in Supplementary Material depicts the protein expression in immunoblot gels.

## Discussion

In summary, our results demonstrate that methylation of a region in the *GPR15* cytokine receptor in response to a common environmental factor, tobacco smoke, is dependent on factors, presumably genetic, which sort with ethnicity. Given the nature of the receptor, it is plausible that it may have implications for understanding ethnicity-related disparities in health outcomes associated with smoking. In addition, these results suggest that this ethnic-sensitive response is not unique and that many additional ethnically sensitive responses to tobacco smoke may exist, suggesting ethnicity-specific pathway to a number of important smoking-related health outcomes.

As compared to better-known chemokine receptors involved with HIV transmission such as CCR5, the cellular roles of GPR15 are less well understood. As a co-receptor for HIV-2, SIV, and some HIV-1 isolates, this chemokine receptor has an established role in facilitating efficient intracellular transport of certain strains of HIV-1, HIV-2, and SIV (20, 21, 29, 30). In addition, increased expression of the receptor is essential for homing of regulatory T-lymphocytes to the gut, and GPR15 signaling plays a key role in moderating gastrointestinal immune tolerance (31). This prior understanding suggests two potential roles for *GPR15* in moderating ethnic-specific differences in susceptibility to both retrovirus infection and subsequent disease course.

Smoking-induced increases in *GPR15* expression may play a role in HIV pathogenesis by increasing the susceptibility of CD4^+^ T cells for some HIV-1 isolates (21, 32). However, secondary to a number of factors, this role may not be consistent in all populations, and before considering potential mechanisms, it is important to understand the limitations of earlier epidemiological studies. At the present time, of the approximately 40,000,000 individuals living with HIV (both HIV-1 and HIV-2), nearly two-thirds are from sub-Saharan Africa, the region from which the majority of African Americans in the United States draw the vast majority of their genetic heritage. Unfortunately, because smoking associates with many other factors that are independently associated with HIV risk, such as IV drug use and risky sex, a direct relationship of HIV infection with smoking is difficult to establish. However, in 2007, Furber and colleagues conducted a meta-analysis of the relationship of smoking to both HIV seropositivity and progression (33). Of the six studies with adequate control for potential confounders that examined the relationship of smoking to HIV seroconversion, five demonstrated a significant relationship between smoking and HIV seroconversion. Significantly, the only study not to demonstrate a significant relationship was a study conducted in 1991 of a cohort that was 89% European American (34). In contrast, all three studies of individuals of African ancestry showed robust independent effects of smoking on seroconversion (35–37). Taken as a whole, these data strongly support the possible existence of ethnic-specific factors in determining elevated HIV seroconversion rates in response to smoking. Differential *GPR15* methylation may play a role in accounting for a portion of that differential vulnerability.

*GPR15*’s role in targeting lymphocytes to the gastrointestinal tract suggests the possibility that smoking may exacerbate HIV-associated enteropathy (30, 38). Optimal gastrointestinal function is facilitated by the presence of a symbiotic gut biome whose composition is regulated, in part, by interactions with regulatory lymphocytes in the intestinal mucosa. Dysregulation of the lymphocyte–biome interplay results in altered epithelial permeability, disruptions of mucosal integrity and at the clinical level both malabsorption and diarrhea. Although the exact mechanisms through which this is accomplished is not yet known, HIV-1 infection directly disrupts mucosal integrity and permeability (39). Therefore, even in the absence of facilitating retrovirus infectivity, by increasing the expression of *GPR15* on lymphocytes and presumably the proportion of lymphocytes segregating to the gut, smoking may be exacerbating gastrointestinal dysfunction. Consistent with this hypothesis is the observation that clinicians routinely recommend smoking cessation to patients with AIDS enteropathy (40). However, even if altered *GPR15* has a role in HIV enteropathy, a generalizable role for smoking-associated altered *GPR15* expression in exacerbating HIV progression is unlikely because in the review of Furber and colleagues, 9 of the 10 high-quality studies reviewed found no relationship between smoking status and HIV progression (33).

The current studies do not provide the exact identity or location of the genetic variation of the smoking-associated changes in *GPR15* expression. Despite the weak, yet convergent data that the rs2230344 SNP is associated with smoking dependent effects in biomaterial from African Americans, the fact that this non-synonymous SNP has no significant effect on methylation in DNA from European Americans demonstrates that this coding polymorphism is not sufficient by itself to trigger smoking-associated changes in gene and protein expression. Instead, these data suggest that other genetic variation co-segregating on the haplotype defined by the rs2230344 SNP acting alone, or in combination with other trans factors, may be responsible for the smoking-associated effect. The latter is a particularly intriguing possibility. Examination of the single exon *GPR15* locus surrounding rs2230344 in the UCSC genome browser (41) demonstrates it to be rich in transcription factor binding sites. However, in order to rigorously test this hypothesis, large-scale studies that incorporate extensive resequencing will be required. Conversely, it is also possible that environmental factors co-segregating with smoking, but differing by ethnicity, may be responsible for the observed effects. Again, testing this hypothesis would require large-scale studies that rigorously interrogate environmental factors.

Combined with other recently published results, our data suggest that the ethnically contextual effects of smoking are not limited to *GPR15* or to comparisons of African Americans with European Americans. Recently, Elliott and associates reported the results of a genome-wide association of DNA from 192 subjects of European and South Asian descents (19). Although they did not specifically examine genetic variation at any locus, they found evidence of ethnic-specific differences at several loci and suggested that ethnic differences in smoking-evoked DNA methylation may provide vital clues to disease pathways that differ by ethnicity. Our results provide specific explication of that assertion with the breadth of the discrepant findings in our analysis suggesting that the vast majority of the highly ranked loci may contain ethnic-specific variation affecting local DNA methylation changes in response to smoking.

Taken in context with other studies, these results give additional credence to prior suggestions that genome-wide genetic searches for the “hidden variance” associated with common complex medical disorders could benefit from incorporation of epigenetic information at the genome-wide level (42, 43). For example, with respect to cardiovascular disease, Breitling and colleagues have shown that methylation status at *F2RL3*, which codes for protease-activated receptor 4 (PAR-4), is strongly associated with mortality in those with coronary heart disease (44, 45). Since coagulation-related outcomes figure prominently in CAD-related mortality, one might expect that variation at this locus might be highly associated with CAD and genome-wide studies. However, review of several genome-wide analyses including one meta-analysis of mixed groups fails to show a significant contribution by genetic variation at this locus (3, 5, 46). Since our current results demonstrate that smoking has a strong ethnically contextual effect on *F2RL3* methylation, and our prior work demonstrates that these smoking-associated effects are strongly correlated with other coagulation factor-related loci, these results strongly suggest that efforts to identify genetic variants responsible for CAD should be both stratified for ethnicity and smoking exposure.

A critical question is how to identify and control for smoking exposure in potentially available, large samples that were not collected with reliable biochemical validation of smoking status. This is not a trivial question because the majority of currently available DNA samples are not well informed with respect to smoking status and high-risk populations have the highest rate of unreliable self-report data. One particularly attractive possibility, given its consistency, may be to use *AHRR* methylation status as a cofactor in analyses to identify smoking exposure. In all populations surveyed today, we and others have repeatedly shown that methylation at *AHRR* residue cg05575921, which is the binding site for a transcriptional enhancer in a key feedback loop in the pathway responsible for metabolizing dioxins and polyaromatic hydrocarbons, is the most consistently differentially methylated residue in smokers. Although there is some evidence that there may be some mild ethnic-dependent effects even at this locus, our unpublished studies of over 2000 individuals of European American, Asian, and African American ancestry indicate that average beta value at cg05575921 in lifetime non-smokers of all ancestries from the United States is approximately 0.91. Unfortunately, use of samples without adequate biochemical validation can inadvertently result in lower values at this locus. This is true even for the current sample taken from the larger, relatively high-risk FACHS population (*n* = 650) in which one fourth of those with markedly positive serum cotinine levels denied the use of tobacco products (unpublished data). But this is not to say that surreptitious smoking might be the only cause of altered basal cg05575921 rates observed in the literature. Therefore, although the use of cg05575921 as a cofactor in genome-wide analyses may improve our power to detect hidden genetic variation, its use is not a perfect solution to the problem posed by smoking-induced differential methylation. However, it still could be a significant improvement.

## Conclusion

In summary, we report that ethnicity has significant effects on genome-wide methylation analyses of the effects of smoking and that genotype at rs2230344 is associated with factors that affect smoking-induced responses. We suggest that ethnically diverse, integrated genetic and methylation approaches may identify genetic variation relevant to risk for smoking-related illnesses and potentially other non-smoking-related common complex disorders.

## Availability of Supporting Data

The Illumina HumanMethylation450 BeadChip data for the male FACHS subjects described in this study are available via Gene Expression Omnibus (GEO) accession GSE59550 while the BD subject data are available via GSE57853.

## Author Contributions

MD, RP, JX, and JS conceived and designed the study and experiments. MD, RP, JX, and JS performed experiments and acquired data. All authors were involved in the analysis and interpretation of the data, drafting and revising the manuscript, read and approved the final version of the manuscript, and agree to be accountable for all aspects of the work.

## Conflict of Interest Statement

The use of methylation at cg05575921 for the quantification of smoking is covered by U.S. Patent 8,637,652 while the use of other loci is patent pending. Dr. Robert A. Philibert is also the Chief Scientific Officer for Behavioral Diagnostics, Inc. None of the other authors have any conflicts to disclose.

## Supplementary Material

The Supplementary Material for this article can be found online at http://journal.frontiersin.org/article/10.3389/fpsyt.2015.00132
