# Supplementary material for: Ethnicity and Smoking-Associated DNA Methylation Changes at HIV Co-Receptor GPR15
Source: Front Psychiatry. 2015 Sep 22;6:132. doi: 10.3389/fpsyt.2015.00132 (PMC4585036; doi:10.3389/fpsyt.2015.00132)
Supplement: Supplementary file 1 [file Image_1.PDF]

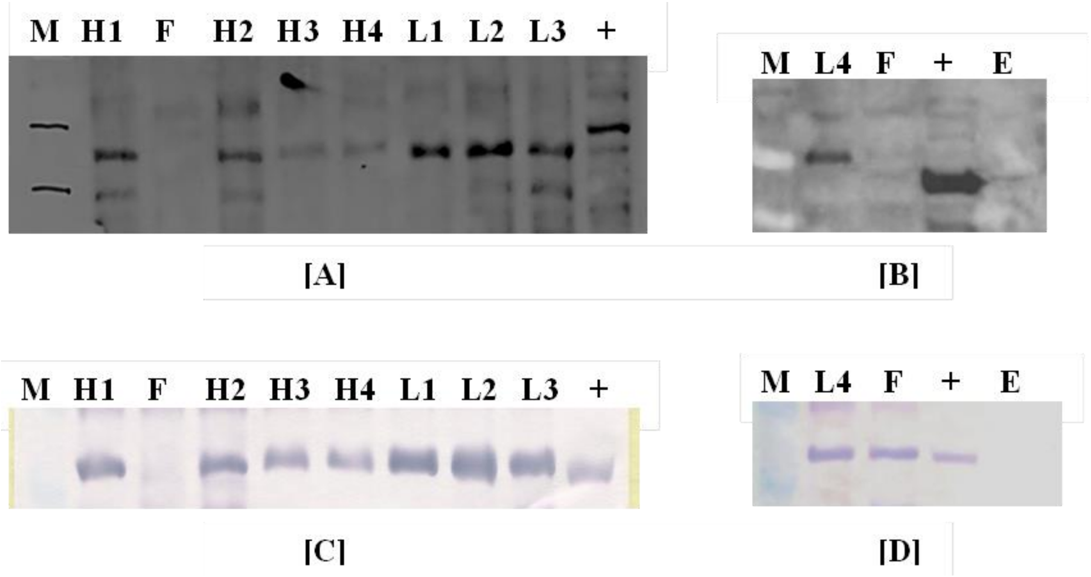

Supplementary File 1. Immunoblot images

Immunoblot image of GPR15 (panels A and B) and actin control (panels C and D) in PBMC lysates. Samples were selected for either high (H) levels or low levels (L) of cg19859270 methylation. The laboratory personnel were blinded to the methylation data. M represents the Precision Plus Protein Dual Color Standard (BioRad, USA). H1 through H4 represent the four samples with the highest cg19859270 methylation (avg beta 0.87 ± 0.01) whereas L1 through L4 represents four samples with the least cg19859270 methylation (avg beta 0.73 ± 0.04). “+” represents the HepG2 cells used as positive control. F represents the two samples that failed and E represents an empty gel lane.
